# SK and Kv4 Channels Limit Spike Timing Perturbations in Pacemaking Dopamine Neurons

**DOI:** 10.1523/ENEURO.0445-22.2023

**Published:** 2023-04-07

**Authors:** Matthew H. Higgs, James A. Jones, Charles J. Wilson, Michael J. Beckstead

**Affiliations:** 1 Aging & Metabolism Research Program, Oklahoma Medical Research Foundation, Oklahoma City, Oklahoma 73104; 2 Department of Neuroscience, Developmental, and Regenerative Biology, University of Texas at San Antonio, San Antonio, Texas 78249; 3 Oklahoma City VA Medical Center, Oklahoma City, Oklahoma 73104

**Keywords:** dopamine, firing, Kv4, PRC, SK, substantia nigra

## Abstract

Midbrain dopamine (DA) neurons are among the best characterized pacemaker neurons, having intrinsic, rhythmic firing activity even in the absence of synaptic input. However, the mechanisms of DA neuron pacemaking have not been systematically related to how these cells respond to synaptic input. The input–output properties of pacemaking neurons can be characterized by the phase-resetting curve (PRC), which describes the sensitivity of interspike interval (ISI) length to inputs arriving at different phases of the firing cycle. Here we determined PRCs of putative DA neurons in the substantia nigra pars compacta in brain slices from male and female mice using gramicidin-perforated current-clamp recordings with electrical noise stimuli applied through the patch pipette. On average, and compared with nearby putative GABA neurons, DA neurons showed a low, nearly constant level of sensitivity across most of the ISI, but individual cells had PRCs showing relatively greater sensitivity at early or late phases. Pharmacological experiments showed that DA neuron PRCs are shaped by small-conductance calcium-activated potassium and Kv4 channels, which limit input sensitivity across early and late phases of the ISI. Our results establish the PRC as a tractable experimental measurement of individual DA neuron input–output relationships and identify two of the major ionic conductances that limit perturbations to rhythmic firing. These findings have applications in modeling and for identifying biophysical changes in response to disease or environmental manipulations.

## Significance Statement

In substantia nigra pars compacta dopamine neurons, pacemaking mechanisms determine the response to an instantaneous synaptic input according to the phase-resetting curve (PRC). Here we measured PRCs of dopamine neurons and determined how they are shaped by small-conductance calcium-activated potassium and Kv4 channels, which regulate pacemaking rate and regularity. We found that both types of channels limit sensitivity to perturbations in firing. Thus, the currents responsible for slow pacemaking also control spike time responses to synaptic input.

## Introduction

Dopamine (DA) neurons in the substantia nigra pars compacta (SNc) and the ventral tegmental area (VTA) are autonomous pacemakers that fire spontaneously in the absence of synaptic input (Sanghera et al., 1984; Grace and Onn, 1989). SNc DA neurons and most VTA DA neurons fire with clock-like regularity in acute brain slices, whose preparation severs most of the synaptic inputs these cells receive *in vivo*. The ionic mechanisms of DA neuron pacemaking have been investigated extensively (for review, see Gantz et al., 2018). The depolarizing currents active between action potentials include persistent voltage-gated and leak sodium currents (Grace and Onn, 1989; Grace, 1991; Puopolo et al., 2007; Khaliq and Bean, 2010; Philippart and Khaliq, 2018; Um et al., 2021), low-threshold calcium currents (Grace and Onn, 1989; Grace, 1991; Yung et al., 1991; Kang and Kitai, 1993a, b; Wilson and Callaway, 2000; Takada et al., 2001; Wolfart and Roeper, 2002; Chan et al., 2007; Puopolo et al., 2007; Philippart et al., 2016), and the hyperpolarization-activated cyclic nucleotide-dependent (HCN) current (Neuhoff et al., 2002). If unopposed, these currents would produce fast burst firing, rapidly leading to sodium channel inactivation and depolarization block (Knowlton et al., 2021).

Slow, regular pacemaking is enabled by calcium-dependent and voltage-dependent potassium conductances. DA neuron pacemaking follows the principles of the classic model of Connor and Stevens (1971), whereby the action potential activates potassium currents producing an afterhyperpolarization (AHP), leading to deinactivation of an A-type (low voltage-activated, inactivating) potassium conductance. The AHP decay is followed by a ramp-like membrane potential (*V*_m_) trajectory largely determined by A-current activation and inactivation. The medium-duration AHP that sets the pacemaking clock after each spike is produced mainly by small-conductance calcium-dependent potassium (SK) channels (Wolfart et al., 2001), which may be activated by calcium influx via L-, T-, and/or N-type channels (Wolfart and Roeper, 2002; de Vrind et al., 2016), and the A-current is generated primarily by Kv4.3 channels (Liss et al., 2001; Haddjeri-Hopkins et al., 2021). Near the end of the interspike interval (ISI), the ramp trajectory is terminated by activation of the spike-generating currents.

The same ionic mechanisms that govern the rate and regularity of pacemaking also determine the responsiveness to synaptic input. However, the pacemaking mechanisms of DA neurons have not been systematically related to the input sensitivity of these cells. Investigators studying input–output properties of pacemaker neurons often hold cells silent using hyperpolarizing current between stimuli, because during pacemaking the response to an input depends on when it arrives within the ISI. However, a pacemaking neuron responds to a synaptic input in a manner different from that to a resting cell (Wilson, 2015). In a resting neuron, small membrane potential perturbations decay with a fixed time constant, and firing occurs only with depolarization sufficient to reach spike threshold. In contrast, a pacemaking neuron responds to every input, excitatory or inhibitory, by changing the ISI length.

The effect of a synaptic input on pacemaking is described by the phase-resetting curve (PRC), which quantifies the change in ISI length as a function of the input phase or the time fraction of the natural interspike trajectory at which the input arrives (Perkel et al., 1964; Reyes and Fetz, 1993; Stiefel and Ermentrout, 2016). For real synaptic inputs, the PRC depends on the input amplitude, the timing within the ISI, and whether it is excitatory or inhibitory. One approach for obtaining a generally applicable PRC for a given cell is to deliver small, brief current pulses at different phases of the ISI. However, it is more efficient to apply a continuous train of small, random-amplitude pulses, or noise, during multiple ISIs (Wilson et al., 2014). By obtaining a PRC for current input and an interspike membrane potential trajectory, the responses of the neuron to synaptic conductances can be predicted. PRCs obtained this way have predicted spike responses of several types of pacemaker neurons (Wilson et al., 2014; Higgs and Wilson, 2017; Simmons et al., 2018, 2020; Morales et al., 2020; Olivares et al., 2022) and other neurons that fire rhythmically when depolarized (Wilson, 2017; Higgs and Wilson, 2019). A previous study measured PRCs of VTA DA neurons using optogenetic stimuli (van der Velden et al., 2020) but did not investigate the ionic mechanisms that determine the PRC.

Here, we determined the PRCs of SNc DA neurons in mouse brain slices using perforated-patch current-clamp recording and noise perturbation. Pharmacological and dynamic clamp experiments were performed to investigate how SK and Kv4 channels affect PRC shapes and overall sensitivity. Our results provide the first detailed description of DA neuron PRCs and identify how they are shaped by these key regulators of pacemaking.

## Materials and Methods

### Animals

All animal procedures were performed in accordance with the Oklahoma Medical Research Foundation animal care and use committee regulations. C57BL/6 mice were bred in-house or purchased from The Jackson Laboratory. Mice were housed in a humidity-controlled and temperature-controlled facility under a 12 h light/dark cycle with food and water available *ad libitum*. Mice were used at ages 3–7 months, and all experimental groups included both males and females.

### Brain slice preparation

Mice were deeply anesthetized with 2,2,2-tribromoethanol (0.25 g/kg) or isoflurane and were killed by decapitation. The brain was removed, the ventral midbrain was blocked, and horizontal slices (200 μm) were cut using a vibrating microtome (model VT1200S, Leica). The cutting solution contained the following (in mm): 2 KCl, 1.2 NaH_2_PO_4_, 26 NaHCO_3_, 11 glucose, 250 sucrose, 7 MgCl_2_, and 0.5 CaCl_2_, bubbled with 95% O_2_ and 5% CO_2_. For some preparations, the cutting solution also contained 3 μm MK-801 hydrogen maleate. After cutting, the slices were transferred to a holding chamber filled with artificial CSF (ACSF) containing the following (in mm): 126 NaCl, 2.5 KCl, 1.2 MgCl_2_, 2.4 CaCl_2_, 1.2 NaH_2_PO_4_, 21.4 NaHCO_3_, and 11.1 glucose, bubbled with 95% O_2_ and 5% CO_2_ and supplemented with 10 μm MK-801. Slices were allowed to recover for at least 2 h before recording and were used up to 10 h after preparation.

### Recording

**Figure 1.**
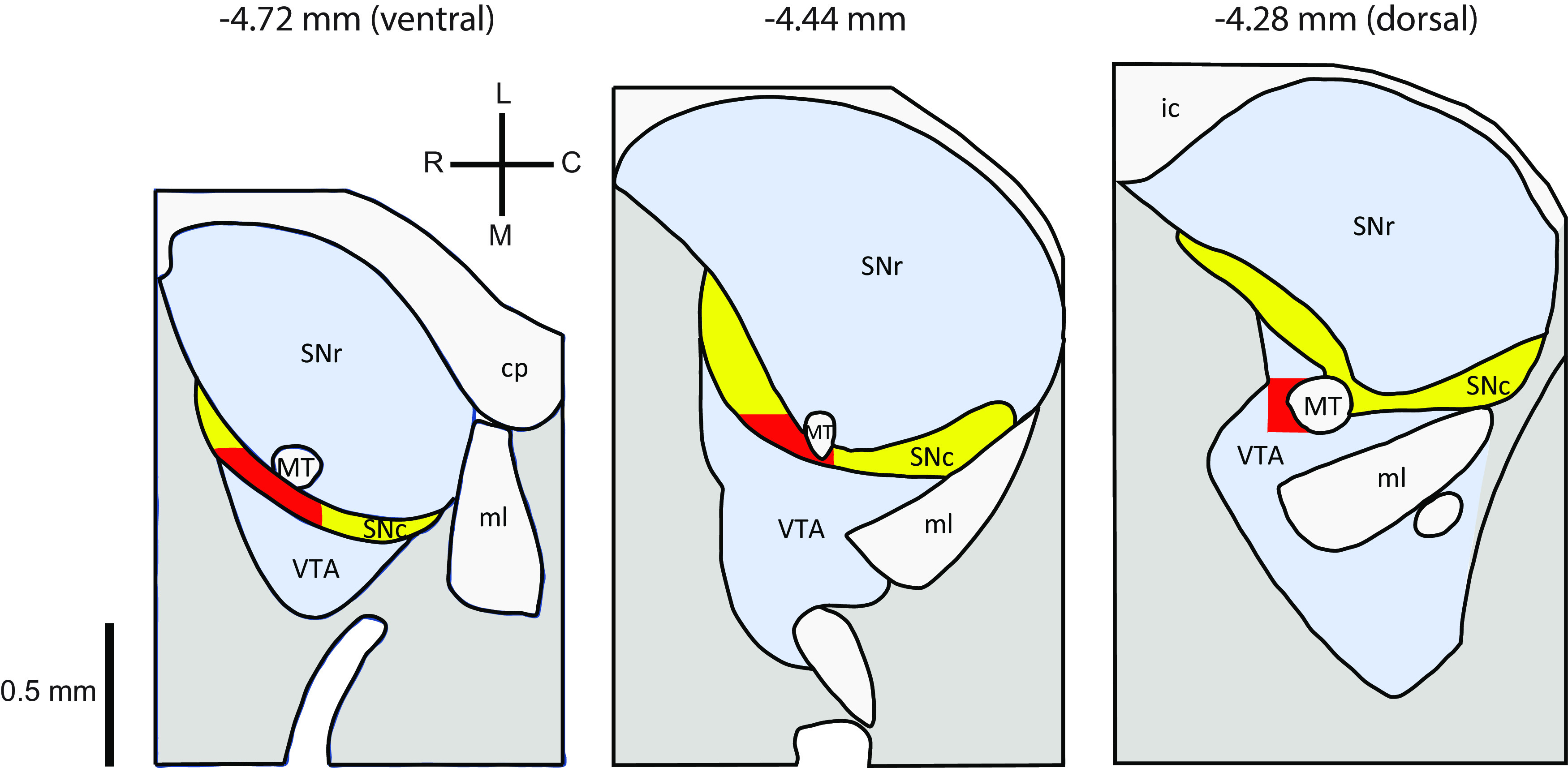
Slice preparation and recording locations. The diagrams are based on the mouse brain atlas of Paxinos and Franklin (2019), from the horizontal sections at −4.72, −4.44, and −4.28 mm relative to bregma, which approximately correspond to the three 200 μm slices in our preparations. The area targeted for recordings is shaded red. According to the atlas, this area lies mostly within SNc but enters the VTA in the most dorsal section. However, DA neurons just medial to the entire ventral–dorsal extent of MT project to the dorsal striatum in addition to the lateral nucleus accumbens (Farassat et al., 2019), suggesting that the targeted area is functionally at the SNc/VTA border. The main measures we report in the Results did not differ significantly between DA neurons from the three slices, so the results were pooled and interpreted as representative of the majority of medial SNc neurons. MT, Medial terminal nucleus of the accessory optic tract; cp, cerebral peduncle; ml, medial lemniscus; ic, internal capsule.

Current-clamp recordings of pacemaking activity were obtained using the gramicidin-perforated patch method. A slice was transferred to the recording chamber and superfused with ACSF maintained at 33–35°C. The area of each slice targeted for recording is shown in Figure 1. Cells were visualized by Dodt contrast microscopy using a microscope (model Eclipse FN1, Nikon) with a 40× objective. Recording pipettes were pulled from borosilicate capillary glass tubing (G150F-4, Warner Instruments). The pipette tip was filled with a solution containing (in mm) 140.5 KCl, 7.5 NaCl, and 10 HEPES, adjusted to pH 7 with KOH, and the pipette was then filled with the same solution supplemented with 1 μg/ml gramicidin-D (MP Biomedicals). The pipette solution was filtered before adding gramicidin. The filled pipettes had resistances of ∼5–6 MΩ. Recordings were obtained using an amplifier (model MultiClamp 700B, Molecular Devices) connected to an analog/digital converter (model InstruTECH ITC-18, HEKA Elektronik) controlled by AxoGraph software. Voltage data were low-pass filtered online (10 kHz) and digitized at 20 kHz. Data collection was started when patch perforation was sufficient to reveal action potentials overshooting 0 mV and to allow adequate bridge balance and capacitance compensation. The access resistance was typically ∼100 MΩ when experiments were started and decreased to ∼50 MΩ over the first 30 min of recording. These changes in access did not affect the efficacy of noise stimulation (described below) based on the coefficient of variation (CV) of the ISIs during noise injection. The membrane potential traces shown were corrected by +10 mV to account for the estimated offset from the true transmembrane potential. This correction was determined from the mean voltage change observed in recordings that broke in suddenly (14 ± 3 mV, *n *=* *20) plus the junction potential for the pipette solution relative to ACSF (−4 mV), which was calculated using the LJPcalc web app.

### Chemicals and pharmacological agents

MK-801 hydrogen maleate, NS309, and AmmTx3 were purchased from Alomone Labs. Apamin was purchased from Echelon Biosciences. Other chemicals were obtained from Sigma-Aldrich.

### Cell type identification

**Figure 2.**
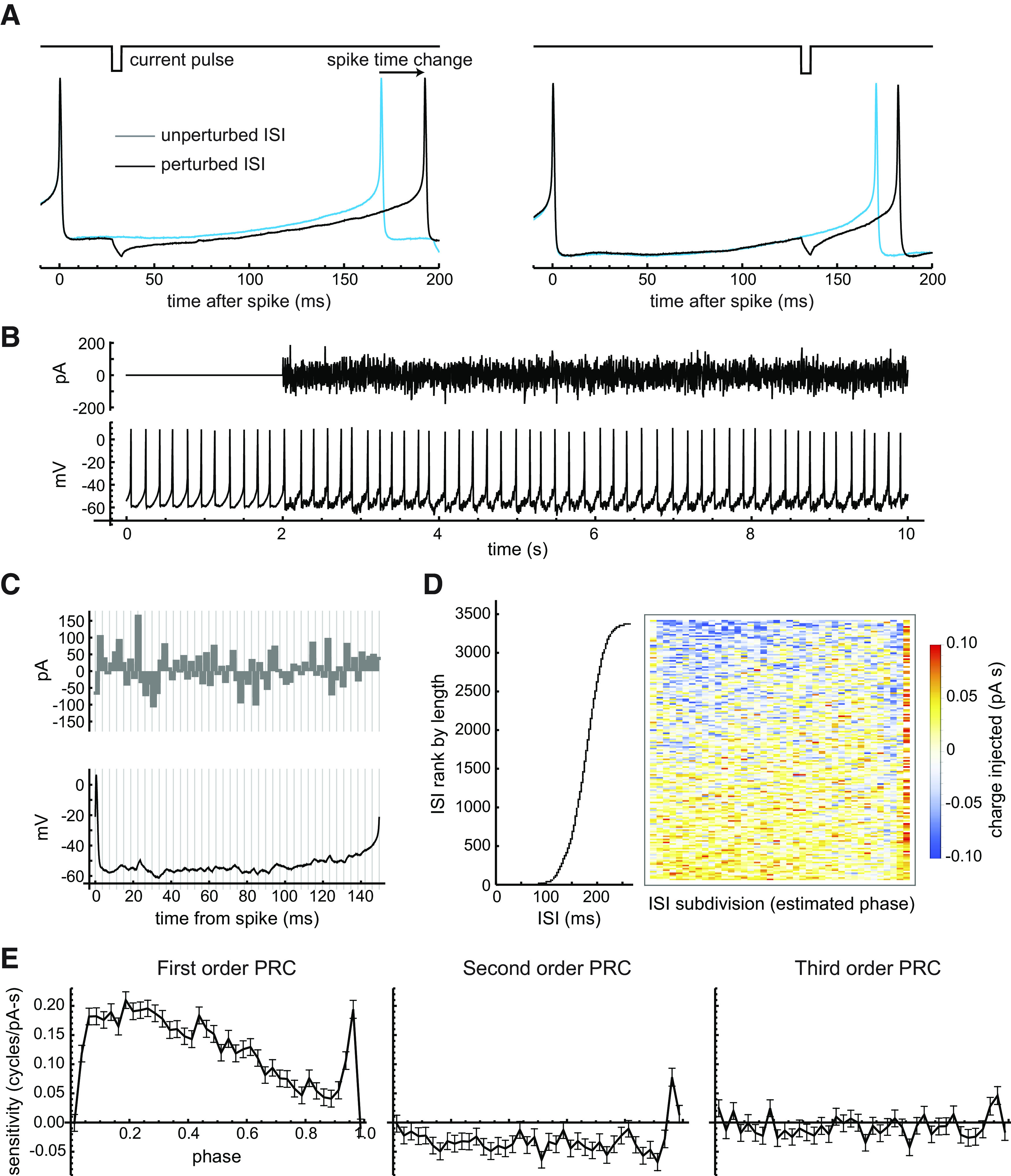
Measuring the PRC. ***A***, Example of phase resetting caused by a hyperpolarizing current pulse. The perturbed ISI (black trace) is compared with the preceding unperturbed ISI (blue trace). The change in spike time caused by the pulse depends on when it is delivered within the ISI. The example DA neuron was not typical but was chosen for illustration because it had larger-than-average spike time changes in response to current pulses. ***B***, Example of noise stimulation used for PRC estimation. ***C***, For analysis, each ISI was divided into 40 equal-length subdivisions (demarcated by gray lines), and the injected charge (shaded region in top graph) was measured. Bottom plot shows the membrane potential perturbations produced by the noise pulses during a single ISI. ***D***, Data for PRC estimation by multiple linear regression. The independent variables are the amounts of charge (pA/s, or pC) injected in the ISI subdivisions, and the dependent variable is the ISI length. For illustration, the charge data were sorted by the ISI length, and the mean values for groups of 20 ISIs were used to produce the color plot, which demonstrates the phase-specific association of injected charge with ISI length. ***E***, First-order, second-order, and third-order PRC of the example neuron, showing the effects of the noise current on the present ISI and the next two ISIs. Error bars indicate the SE of the estimate for each PRC value. Consistent with the effects of single current pulses shown in ***A***, the first-order PRC of the example neuron showed higher sensitivity to injected charge at early versus late phases, until the sensitivity rose to a sharp peak near the end of the ISI.

All data analysis was performed in Mathematica (Wolfram Research). Putative DA and GABA neurons were distinguished by spike width at half-height, taken halfway between the apparent spike threshold (at 10 mV/ms) and the peak of the action potential. Spike width was measured from averaged action potentials (aligned on the rising phase) recorded during unperturbed pacemaking and was >0.6 ms for all cells identified as DA neurons and <0.6 ms for all cells identified as GABA neurons. In addition, the GABA neurons had natural firing rates above the range found in DA neurons (Fig. 2). Other physiological properties including the presence or absence of a voltage sag during hyperpolarizing current steps and a rebound delay after hyperpolarizing steps were consistent with these criteria.

### Phase-resetting curve measurement

PRCs were determined by noise stimulation and multiple-regression analysis. The procedure was designed to estimate as closely as possible the infinitesimal PRC, or the PRC for a vanishingly small input which, by definition, applies equally to depolarizing and hyperpolarizing input. The major advantages of this method over standard PRC measurements using single-stimulus pulses are time efficiency and the absence of a response-independent bias at late phases, as demonstrated by the absence of a spurious PRC when ISI data are analyzed with respect to a noise stimulus that was not actually applied. For PRC measurement, small-amplitude, zero-mean noise stimuli were used to produce small, bidirectional perturbations of the ISIs. The standard protocol included 80 episodes of 10 s duration, each with a 2 s baseline followed by 8 s of noise. The noise was made up of contiguous 2 ms square current pulses, each having an amplitude drawn from a Gaussian distribution with an SD of 50 pA. For GABA neurons, which are more sensitive, we used a weaker noise stimulus with 0.5 ms pulses and an SD of 40 pA to avoid excessive changes in their ISI lengths, thereby improving the estimation of phase as the time fraction of the ISI traversed. It was previously demonstrated that this PRC estimation method produced similar results for a wide range of noise levels, so it was not necessary to adjust the stimulus based on the responses of each individual neuron (Wilson et al., 2014). The noise waveforms were created offline using Mathematica and were delivered to neurons using AxoGraph.

The data were analyzed by separating each ISI into 40 equal-length bins, which were assumed to represent equal ranges of phases spanning the firing cycle (represented by values from 0 to 1). The injected charge (*Q_i,j_*) was measured for each ISI (*i*) and each bin (*j*), and a multiple-regression analysis was performed to obtain the PRC value for each bin. The dependent variable for the regression is the relative ISI length (*T_i_*/*T*_avg_), and the regression coefficients provide the PRC value (*Z_j_*) for each bin and the value *T*_0_/*T*_avg_, where the estimated unperturbed ISI, *T*_0_, may differ from the average ISI during noise perturbation, *T*_avg_, if the average noise stimulus arriving during the ISI has a nonzero projection on the PRC. The regression equation is as follows, where *ε_i_* is the residual error for ISI *i*:

(1) $$\frac{T_{i}}{T_{\text{avg}}}=\frac{T_{0}}{T_{\text{avg}}}-\overset{n}{\underset{j=1}{\sum }}Z_{j}\;Q_{i,j}+\varepsilon_{i}.$$

The negative sign of the sum indicates the convention that positive *z*-values represent ISI shortening by positive charge injection. For current in picoamperes and time in seconds, the PRC values have units of cycles pA/s. The PRC analysis was extended beyond the initial ISI to include second-order and third-order PRCs by analyzing the charge injected in bins spanning three ISIs, where *T_i_* in Equation 1 then represents the last of the three ISIs. The extended PRC analysis provides three sets of *n* PRC values, which represent the effect of an input on the present ISI (first-order PRC), the following ISI (second-order PRC), and the ISI after that (third-order PRC).

### Dynamic clamp

Dynamic current clamp was used to add an artificial AHP conductance to recorded DA neurons. The current produced by the AHP conductance was calculated in real time and added to the noise stimuli described above using RTXI (Real-Time eXperimental Interface software; www.rtxi.org) running under a modified Linux operating system. Data input/output was controlled by an analog-to-digital board (model PCIe-6251, National Instruments). The artificial AHP conductance was a spike-triggered, simulated potassium conductance (*E*_rev_ = −90 mV) designed as a simplification of the medium AHP conductance produced by SK channels. The artificial AHP conductance was increased by 4 nS upon detection of each action potential at an upward crossing of −10 mV, and decayed with a time constant of 50 ms.

### Data and statistics

The data reported in the text are the mean ± SD, and error bars on graphs indicate the SEM unless indicated otherwise. The significance of differences was evaluated by paired or unpaired *t* tests, or by a Mann–Whitney *U* test when samples failed a test for normality. The significance of correlations was tested by the Pearson correlation test. Differences were considered significant when *p *<* *0.05.

## Results

### Measuring the PRC

The primary effect of a brief synaptic input to a pacemaking neuron is a change in length of the ISI during which that input arrives (Fig. 2*A*), which shifts the phase of the following spike train. The PRC measures the sensitivity of the phase shift of the neuron to current arriving at each phase of the ISI. The sensitivity is defined as the relative ISI change per unit of charge injected, in cycles/pA-s or cycles/pC. These units are equivalent to those for the slope of the firing frequency–current curve, and thus represent sensitivity in the same sense but on a finer timescale. The PRC provides a first-order description of the neuron’s input–output relationship (Stiefel and Ermentrout, 2016).

Although the PRC is traditionally defined by the effects of single, brief inputs, noise stimuli (Fig. 2*B*) provide a much more efficient method for PRC estimation. Using noise stimuli, hundreds of thousands of stimulus pulses comprising the noise can be delivered during a stable recording, providing more precise PRC estimates despite the natural variation of ISI length caused by intrinsic ion channel noise. Assuming the neuron’s average phase trajectory during the noise-perturbed ISIs is approximately linear, we can estimate phase as the time fraction of the present ISI traversed and determine the PRC by multiple linear regression, where the independent variables represent charge injected in subdivisions of each ISI (i.e., phase bins; Fig. 2*C*). and the dependent variable is ISI length (see Materials and Methods). The charge injection data for an example analysis are illustrated by the density plot in Figure 2*D*. In the example, it is apparent that shorter ISIs were associated with greater positive charge injected, and this association varied according to the phase of the ISI at which the charge was delivered. The PRC obtained from this cell is shown in Figure 2*E* (left panel). The example PRC has a distinctive shape, with a broad body rising to a peak at a phase of ∼0.2 and then declining, followed by a sharp peak at late phase. This shape indicates that the neuron was relatively sensitive to input arriving at early phases, then lost sensitivity, and then gained sensitivity near the end of the ISI. The variation of sensitivity across the firing cycle represented by the PRC determines the spike-time responses of the neuron to time-varying inputs.

In addition to altering the present ISI, a brief input might also change subsequent ISIs by producing changes in ion channel gating that are not reset by the next action potential or by altering the carryover of currents activated by previous spikes. The effects on the lengths of the following two ISIs can be characterized by second-order and third-order PRCs analogous to the first-order PRC described above (Canavier and Achuthan, 2010). We measured the first-, second-, and third-order PRCs by extending the analysis to include, as independent variables, the charge injected in a set of bins spanning three ISIs (Fig. 2*E*). In the example cell, we see that the second-order PRC had a distinct shape that was neither a replica nor a mirror image of the first-order PRC, suggesting that it did not arise entirely from the primary effect on ISI length. Instead, the second-order PRC was largely negative (showing an increase in the second ISI by depolarizing input, consistent with typical spike frequency adaptation) but had a sharp positive peak at the end. Thus, depolarizing input arriving only at the latest phases shortened two successive ISIs. The mechanism of this effect is not known, but it is reminiscent of the observations of Kang and Futami (1999), who showed that suprathreshold activation of subthalamic excitatory inputs to SNc DA neurons could generate a short-latency action potential without producing a large change in the next spike time, thereby shortening two ISIs. The third-order PRC was much smaller in amplitude, approaching the noise level of the measurement, but appeared to resemble the second-order PRC. Thus, the major effects of brief current input appeared to be restricted to two ISIs.

### DA and GABA neuron PRCs

**Figure 3.**
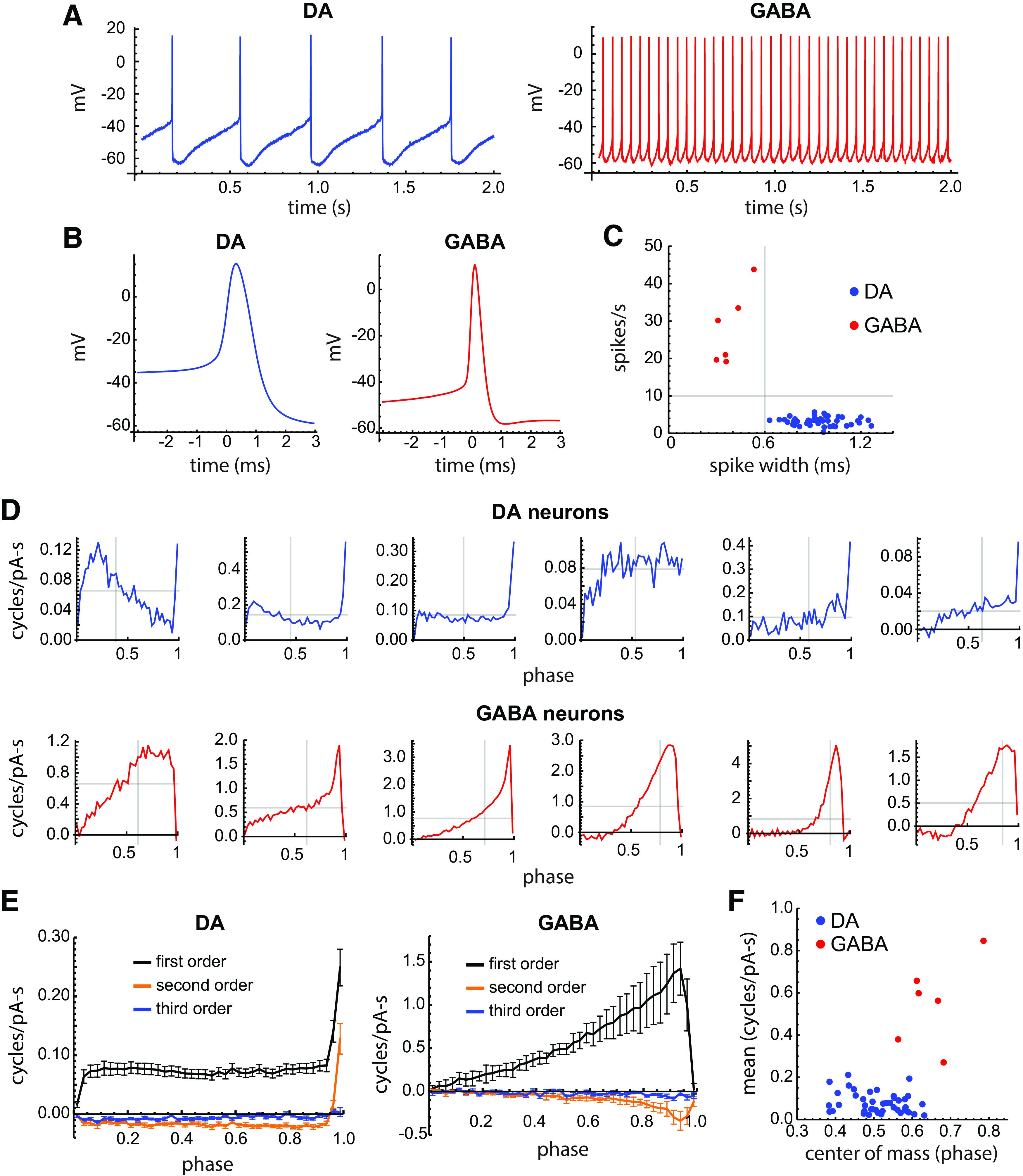
DA and GABA neuron PRCs. ***A***, Typical examples of spontaneous firing in DA and GABA neurons. ***B***, Average action potential waveforms in the example cells. ***C***, Scatter plot of mean spontaneous firing rate versus spike width at half-height for DA neurons and GABA neurons. Gray lines indicate a spike width of 0.6 ms and a firing rate of 10 Hz. The DA and GABA neurons in this sample could be separated by either criterion. ***D***, Example primary PRCs from individual DA neurons (top row) and GABA neurons (bottom row), each sorted by their center of mass (left to right). The gray lines superimposed on each PRC indicate the center of mass (vertical line) and the mean value (horizontal line). ***E***, Average first-order, second-order, and third-order PRCs of DA and GABA neurons. Error bars indicate the SEM. ***F***, Scatter plot of the mean PRC value versus the center-of-mass phase for the first-order PRCs of DA neurons (blue symbols) and GABA neurons (red symbols).

Using the methods described above, PRCs were obtained from 43 SNc DA neurons and 6 GABA neurons encountered in the same area (in SNc or at the SNc border with pars reticulata). The two cell types were segregated by their spontaneous firing rates and spike widths (Fig. 3*A–C*). PRCs of individual DA neurons were heterogeneous in amplitude and shape, illustrated by the six examples shown in Figure 3*D* (top row). As simple measures of PRC properties, we determined the mean amplitude of the primary PRC across the 40 phase bins, and the center-of-mass phase. For the center-of-mass measurement, we excluded the first two bins (representing phases of 0–0.05) and the last two bins (phases of 0.95-1) to remove the final peak, the size of which appeared to be largely independent from the main body of the PRC. A center of mass of <0.5 indicates a left-weighted PRC (higher sensitivity at early vs late phases), and a center of mass >0.5 indicates a right-weighted PRC. The heterogeneity of DA neuron PRCs indicates that these cells would spike at different times even if they were to receive identical synaptic input, and even if their natural pacemaking rates were the same.

PRCs of GABA neurons were measured in the same manner, but a weaker noise stimulus (pulse length, 0.5 ms; SD, 40 pA) was used to avoid excessive perturbation of their ISI lengths, which might compromise the assumption that phase can be estimated as the time fraction of the ISI traversed. The GABA neuron PRCs are plotted in Figure 3*D* (bottom row), sorted by the center of mass. It is apparent that the GABA neurons had higher overall sensitivity (indicated by the mean PRC amplitude) than the DA neurons (*p *=* *0.00008, Mann–Whitney *U* test), and their PRCs were weighted toward later phases (*p *=* *0.00002, *t* test), showing increasing sensitivity across most of the ISI. Two of the GABA neuron PRCs had a small negative lobe, indicating that depolarizing input at early phases lengthened the ISIs, and so would be classified as type 2 (Hansel et al., 1995). PRCs with similar shapes, but generally without a negative lobe, were reported previously in studies of substantia nigra pars reticulata neurons (Simmons et al., 2018, 2020).

The average first-order, second-order, and third-order PRCs of DA and GABA neurons are shown in Figure 3*E*. Although few individual DA neurons had flat PRCs, the average DA neuron PRC was nearly flat except for the first point (representing phases of 0–0.025) and the last two points (phases of 0.95–1). In contrast, the average GABA neuron PRC sloped upward across most of the firing cycle before falling toward zero in the last two points. This final falloff is expected because neurons generally lose sensitivity as the action potential begins to initiate, but it is likely not resolved for the DA neurons because each of the 40 bins into which the ISI was divided represented a much longer time for these slower-firing cells. The second-order PRCs of GABA neurons also differed from those of DA neurons, being entirely negative in sign, indicating that depolarizing input always lengthened the following ISI. The third-order PRCs nearly vanished for both cell types, indicating that the major effects of brief current inputs were limited to two ISIs.

The measures of DA and GABA neuron first-order PRCs are compared in Figure 3*F*, showing the lower overall sensitivity and more symmetrical PRC shapes of DA neurons. This comparison indicates that the different pacemaking mechanisms present in the two cell types produce striking differences in input sensitivity and PRC shapes, in addition to the well established difference in autonomous firing rates.

### Membrane potential responses underlying the PRC

In theory, a pacemaking neuron repeatedly traverses a closed trajectory, or limit cycle, in a state space defined by the *V*_m_ and numerous other state variables (i.e., ion channel gating variables) that are not experimentally observable. We may, however, gain insight about the mechanisms that shape the PRC by observing how a perturbation at a given phase changes the *V*_m_ trajectory leading up to the next action potential. For example, voltage perturbations in pacemaking subthalamic neurons do not relax as they would in a resting neuron. Rather, a depolarizing input essentially advances the cell to a later point in the natural trajectory (Farries et al., 2010).

**Figure 4.**
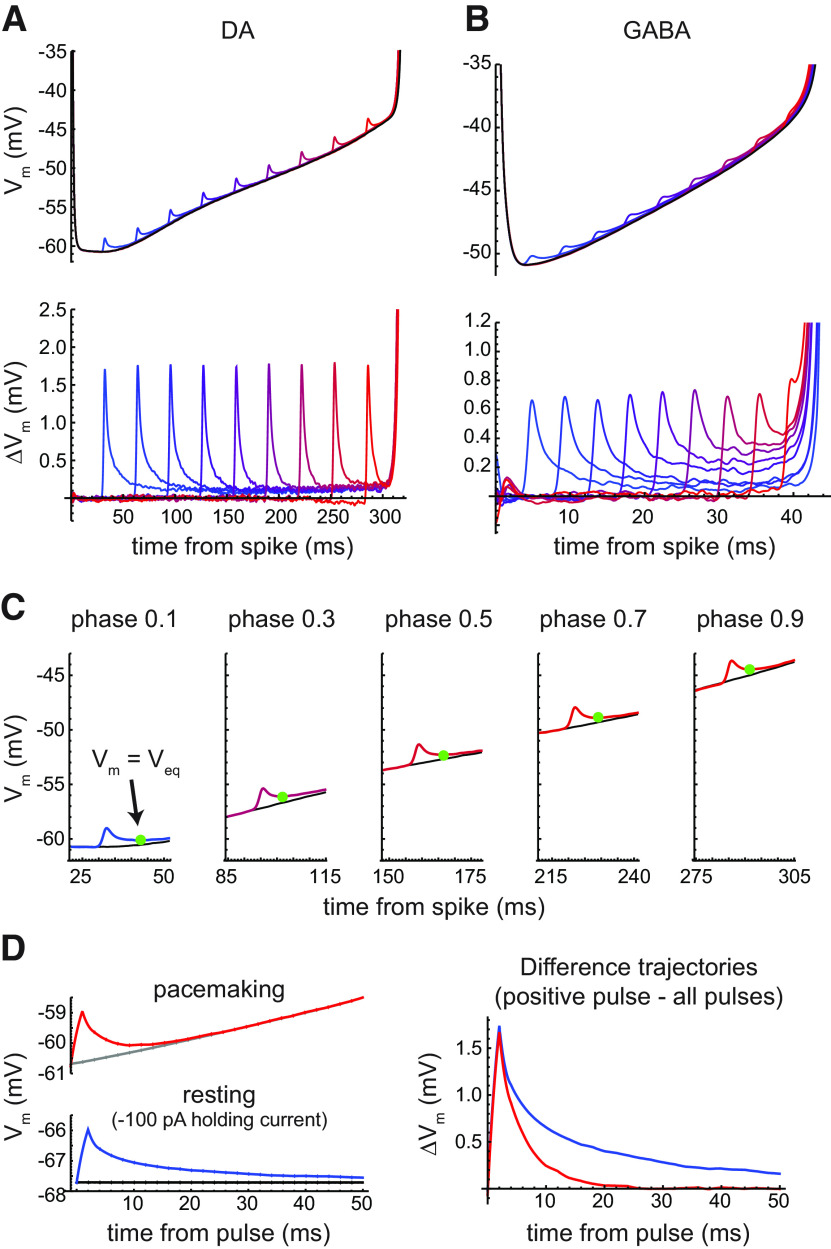
Membrane potential trajectory responses to current pulses within the noise stimulus. The interspike *V*_m_ trajectories shown are grand averages for the entire sample of DA or GABA neurons. ***A***, Top, Perturbed trajectories of DA neurons, each selected for the presence of a positive current pulse at a specified phase (0.1–0.9, color coded blue to red), compared with the average of all trajectories during noise stimulation (black). Bottom, Difference trajectories obtained by subtracting the overall average trajectory from each perturbed trajectory. The strong relaxation of the initial *V*_m_ perturbations led to very small changes in ISI length despite initially substantial depolarizations. ***B***, Perturbed trajectories and difference trajectories of GABA neurons. Compared with the DA neuron data, these responses showed less relaxation, particularly in the second half of the ISI, which was associated with larger relative changes in ISI length for a given initial *V*_m_ change. The magnitude of *V*_m_ change is not directly comparable to the DA neuron data because of the smaller, briefer noise pulses applied. ***C***, Estimation of the moving equilibrium potential (*V*_eq_) from the time course of *V*_m_ relaxation after positive perturbations in DA neurons (same data as in ***A***). *V*_eq_ was estimated as the value of *V*_m_ at which d*V*_m_/d*t* reached zero before the trajectory resumed its upward course (green symbol). These data suggest that *V*_eq_ differed by only a fraction of a millivolt from the natural trajectory across most of the firing cycle. ***D***, Example of difference in *V*_m_ relaxation between pacemaking (no holding current) and resting (−100 pA holding current). The top left traces show the average *V*_m_ responses after current pulses within the noise stimulus delivered during pacemaking, excluding pulses in the first 10 ms and the last 50 ms of each ISI; the red trace is the average for positive pulses, and the gray trace is the average for all pulses. The bottom left traces show the average *V*_m_ responses after current pulses within the noise stimulus delivered while the cell was held at rest; the blue trace is the average for positive pulses, and the black trace is the average for all pulses. The right graph shows the difference trajectories during pacemaking (red) and at rest (blue). The slower relaxation of *V*_m_ perturbations seen at rest indicates that the fast relaxations shown in ***A*** and ***C*** are not artifactual, but instead depend on membrane conductances activated during pacemaking, which cause the total membrane conductance to exceed the resting level.

A major obstacle to measuring small perturbations of the *V*_m_ trajectory is that interspike trajectories vary because of intrinsic ion channel noise and spontaneous synaptic input. To overcome this, we measured the average effects of thousands of perturbations produced by the noise stimuli used to determine the PRC. To minimize voltage artifacts caused by imperfect bridge balance and capacitance compensation, all the data used for this analysis were subjected to *post hoc* bridge balance recorrection and capacitance artifact removal. For each perturbation phase examined, we determined whether the noise pulse being delivered at that fraction of the ISI was positive or negative. The *V*_m_ trajectories for all ISIs with a positive noise pulse at the specified phase (approximately half of the ISIs) were sampled at 1000 equally spaced time points spanning the ISI, and the times relative to the first spike and the *V*_m_ values were averaged. This analysis was performed for a set of perturbation phases (0.1–0.9 at increments of 0.1) across the firing cycle. To reduce noise further, grand average perturbed trajectories were obtained from the entire sample of neurons for every perturbation phase, and these were compared with the grand average of all trajectories (Fig. 4*A*, top).

To examine the trajectory changes, the overall grand average trajectory was subtracted from each perturbed trajectory to produce difference trajectories (Fig. 4*A*, bottom). At most phases, the initial *V*_m_ change caused by the pulse relaxed almost but not quite completely to the natural trajectory, leaving a small offset that was evidently responsible for the small change in spike time produced. In contrast to the DA neurons, GABA neurons showed less relaxation of the *V*_m_ changes, particularly at late phases (Fig. 4*B*). These data suggest that the higher overall sensitivity and right-weighted PRCs of GABA neurons arise from interspike *V*_m_ trajectories that retain more of the initial deflection from the natural trajectory, particularly in the later portion of the ISI.

These data indicate that although DA neurons are integrator like in the sense of having, on average, relatively constant sensitivity across the ISI, they integrate only a small fraction of their current input, discarding the rest to return near their natural trajectory. On a short timescale, they respond to *V*_m_ perturbations as though they were at a stable equilibrium potential although they are actively pacemaking. After depolarizing current pulses, the equilibrium potential can be estimated as the value of *V*_m_ where the slope of the trajectory (d*V*_m_/d*t*) falls to zero before resuming the slow depolarization toward the next action potential (Fig. 4*C*). This estimate is not exact because d*V*_m_/d*t* also depends on axial current flow among the compartments of the neuron. However, qualitatively, the observation of fast *V*_m_ relaxations to an apparent equilibrium potential near the natural trajectory suggests that the phase of a perturbed DA neuron (as defined by the time to its next spike in the absence of further input) does not depend primarily on the present value of *V*_m_. Instead, the phase must depend on slowly varying ion channel gating variables that set the equilibrium potential at each phase and are altered only slightly by brief changes in *V*_m_. Some of these variables are considered later in the Results.

To alleviate concern that the fast *V*_m_ relaxations are in some way artifactual and might not result from the membrane conductances active during pacemaking, we perturbed three DA neurons with noise stimuli during pacemaking and while the neuron was held at rest with a negative holding current (−100 pA). An example cell is illustrated in Figure 4*D*. In the case of pacemaking, the *V*_m_ responses were averaged for positive pulses and for all pulses at all phases of the ISI excluding the first 10 ms and the last 50 ms. At rest, the responses were averaged for positive pulses and for all pulses across each episode of noise perturbation. This analysis demonstrated that *V*_m_ perturbations relaxed much faster during pacemaking than while the neuron was held at rest. Thus, the fast relaxations observed during pacemaking result from increased membrane conductance relative to the resting state, which pulls *V*_m_ toward a moving equilibrium potential.

### Effects of firing rate on DA neuron PRCs

The effect of a brief input on the phase of a pacemaking neuron may be expected to vary according to the firing rate of the cell. If the change in cycle duration caused by a given charge injection at a particular phase were constant, the PRC, expressed in cycles of phase advance per unit charge injected, would scale in proportion to the firing rate. However, this type of scaling is seldom observed in real neurons or in their biophysical models. Instead, the relationship between firing rate and the PRC depends on the unique, rate-dependent pacemaking dynamics of each neuron, which vary substantially among neurons of different types. PRCs of some neurons are relatively insensitive to rate changes, whereas the PRC sizes and shapes of other cell types are strongly rate dependent (Gutkin et al., 2005; Tsubo et al., 2007; Phoka et al., 2010; Wilson et al., 2014; Couto et al., 2015; Simmons et al., 2018).

**Figure 5.**
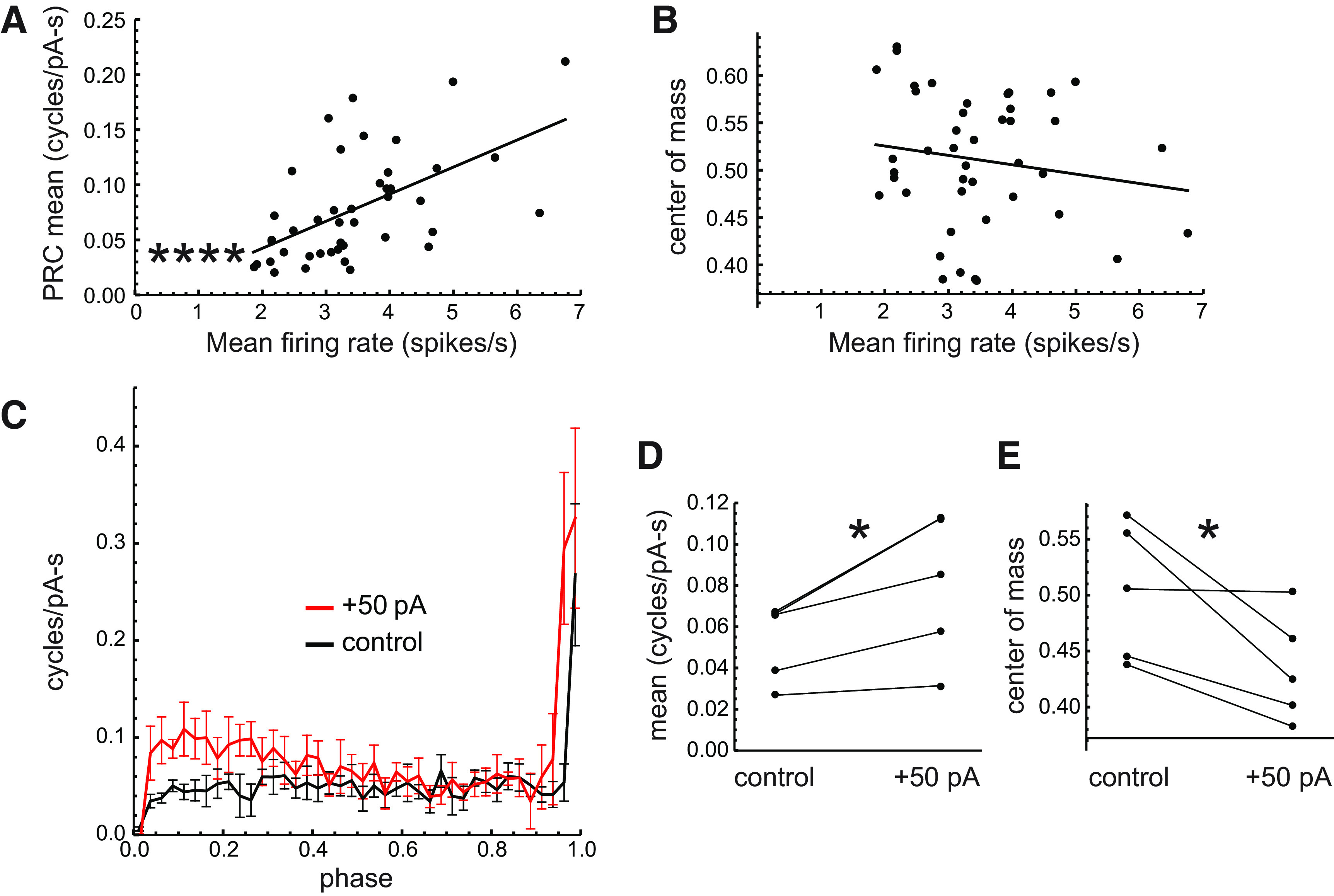
Effects of firing rate on DA neuron PRCs. ***A***, Mean PRC value versus mean firing rate during noise perturbation for each cell. The superimposed line is the linear regression. Asterisks indicate *p *<* *0.0001 (Pearson correlation test). ***B***, PRC center of mass versus mean firing rate. ***C***, Average PRCs of five DA neurons tested with alternating episodes of noise stimulation with and without a +50 pA direct current added to the noise. The error bars indicate SEM. ***D***, Mean value of each PRC without and with the direct current (*p *<* *0.05, paired *t* test). ***E***, Center of mass of each PRC without and with the direct current.

To examine the relationship between firing rate and the PRC in our sample of DA neurons, we plotted the mean sensitivity and PRC center of mass against the mean firing rate during noise perturbation (Fig. 5*A*,*B*). These data show that the mean sensitivity was strongly correlated with the mean rate (*r *=* *0.56, *p *=* *0.00002^a^, Pearson correlation test; Table 1), whereas the center of mass showed no significant relationship with rate (*r* = −0.16, *p *=* *0.31).

**Table 1:** Statistics

	Figure	Graph	Data structure	Type of test	*p*-Value	Power (95% CI of difference)
a	5	*A*, *		Pearson correlation	0.00002	
b	5	*D*, *	Normal distribution	Paired *t* test	0.03	0.011–0.043
c	5	*E*, *	Normal distribution	Paired *t* test	0.04	−0.114 to −0.023
d	6	*C*, *	Normal distribution	Paired *t* test	0.002	−1.90 to −0.77
e	6	*D*, *	Normal distribution	Paired *t* test	0.02	−0.025 to −0.004
f	6	*G*, *	Normal distribution	Paired *t* test	0.006	−0.082 to −0.025
g	6	*H*, *	Normal distribution	Paired *t* test	0.003	0.073–0.205
h	7	*B*, *	Normal distribution	Paired *t* test	0.04	0.57–2.64
i	7	*F*, *	Normal distribution	Paired *t* test	0.03	0.058–0.236
j	8	*C*, *	Normal distribution	Paired *t* test	0.0002	−1.26 to −0.66
k	8	*D*, *	Normal distribution	Paired *t* test	0.03	−0.035 to −0.007
l	8	*F*, *	Normal distribution	Paired *t* test	0.013	−0.093 to −0.023
m	9	*B*, *	Normal distribution	Paired *t* test	0.04	−0.396 to −0.064
n	9	*D*, *	Normal distribution	Paired *t* test	0.03	0.42–1.96
o	9	*G*, *	Normal distribution	Paired *t* test	0.046	0.013–0.086
p	9	*H*, *	Normal distribution	Paired *t* test	0.03	0.037–0.192

To examine the effect of changing the firing rate on the PRC in individual DA neurons, we alternated episodes of noise stimulation with and without a superimposed +50 pA direct current injection, which increased the mean firing rates from 3.11 spikes/s (SD, 1.07) to 5.56 spikes/s (SD, 1.63; *n *=* *5 cells). The average PRCs with and without the added current are shown in Figure 5*C*, and the mean PRC values and centroids of each cell without and with the direct current are compared in Figure 5, *D* and *E*. Raising the firing rate by direct current injection increased the mean sensitivity measured by the PRC (control: 0.053 cycles/pA/s; SD, 0.019; +50 pA: 0.080 cycles/pA/s; SD, 0.035; *p *=* *0.03^b^, paired *t* test). In contrast to the effects of natural heterogeneity of pacemaking rates, raising the firing rates of individual neurons also changed the PRC center of mass (control: 0.50; SD, 0.06; +50 pA: 0.43; SD, 0.05; *p *=* *0.04^c^, paired *t* test). These results suggest that the PRC shape may be affected differently by the natural mechanisms of pacemaking rate heterogeneity than by a direct current. The mechanisms responsible for the rate dependence of the PRC are not known, but some of the interactions may be explained by the conductances discussed below.

### Effects of SK channels on DA neuron firing activity and PRCs

Although inward sodium and calcium currents are essential for pacemaking, they are not sufficient to produce the slow firing and clock-like regularity of SNc DA neurons in brain slices. The main slow variables that govern the pacemaking clock are the ones that determine the activation and inactivation of potassium conductances. SK channels produce a major component of the AHP and are essential for the slow rate and regularity of pacemaking in SNc DA neurons (Gu et al., 1992; Wolfart et al., 2001; Ji et al., 2009; Deignan et al., 2012; Iyer et al., 2017). SK channels in DA neurons respond to widespread increases in intracellular calcium rather than being coupled tightly to sites of calcium influx (Wilson and Callaway, 2000), and, thus, the time course of intracellular calcium elevation is one of the critical slow variables that governs pacemaking.

**Figure 6.**
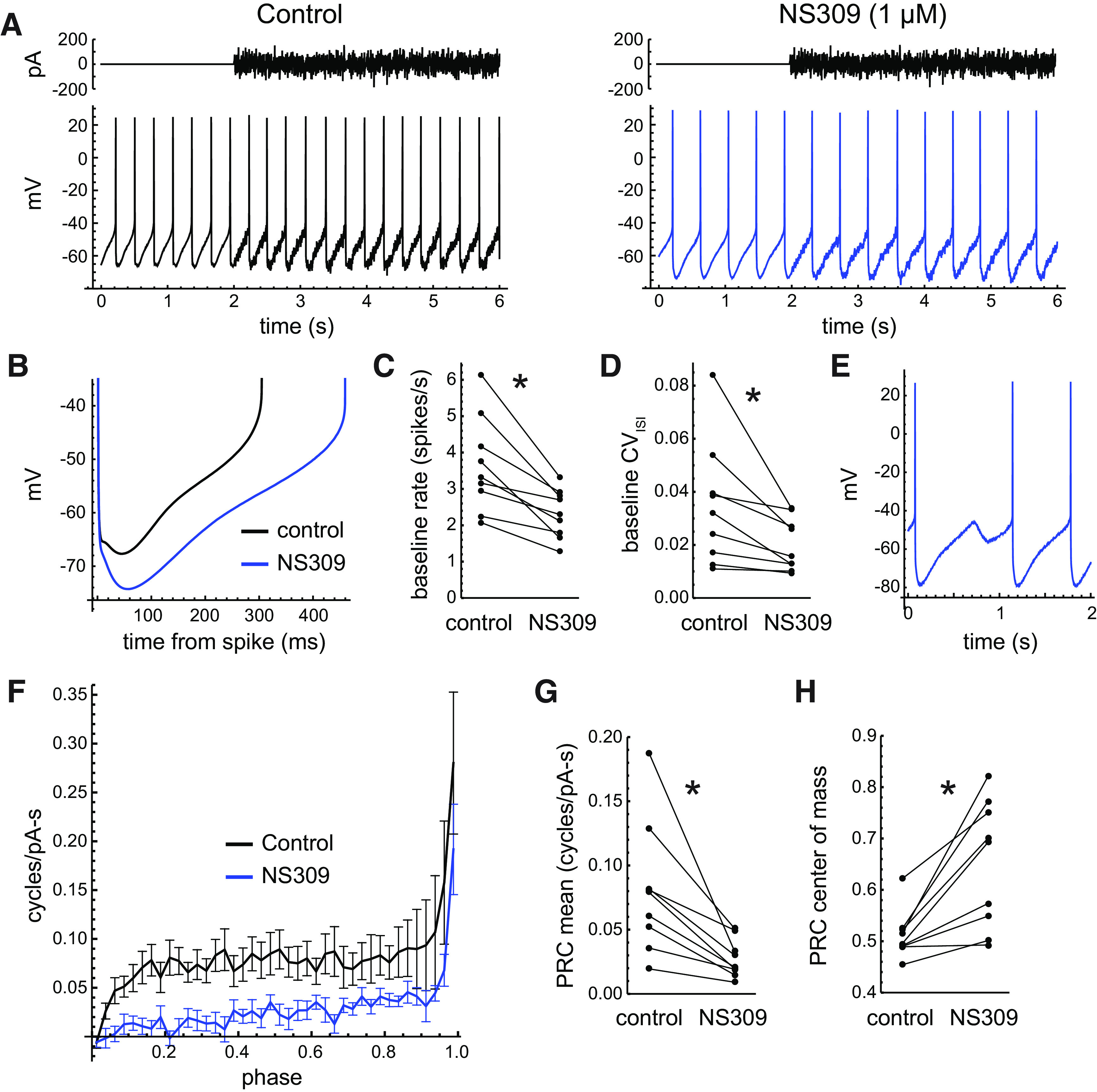
Effects of the SK channel positive modulator NS309 on DA neuron firing activity and PRCs. ***A***, Pacemaking of an example neuron in control ACSF and in the presence of 1 μm NS309. The noise stimulus (top) was identical for the two episodes shown. ***B***, Average baseline interspike *V*_m_ trajectories of the example cell in control ACSF and with NS309, showing the enhancement of the AHP. ***C***, Mean baseline firing rates in control ACSF and with NS309 (*p *<* *0.05, paired *t* test). ***D***, CV of baseline ISIs in control ACSF and with NS309. These data, and the mean firing rate and PRC data, were limited to the periods of NS309 application when pacemaking remained regular. ***E***, Example of a missed beat, or spontaneous transition to subthreshold oscillation, in the example neuron later during the application of NS309. Data showing this phenomenon were not used for PRC analysis. ***F***, Average PRCs in control ACSF and with NS309. Error bars indicate SEM. ***G***, Mean PRC values in control ACSF and with NS309 (*p *<* *0.05, paired *t* test). ***H***, PRC center of mass in control ACSF and with NS309.

To investigate the effects of SK channels on DA neuron PRCs, we first applied the SK/IK (intermediate-conductance KCa) channel positive modulator NS309 (Ji et al., 2009; Christophersen and Wulff, 2015) at a concentration of 1 μm. Because NS309 was dissolved in DMSO, the same amount (0.1%) was added to the control ACSF. An example of the effect of NS309 on firing at baseline and during noise perturbation is shown in Figure 6*A*. NS309 caused a deepening of the AHP, demonstrated by the average interspike trajectories of the example cell (Fig. 6*B*). NS309 lowered the firing rates of all the neurons tested (control: 3.66 spikes/s; SD, 1.32; NS309: 2.32 spikes/s; SD, 0.67; *n *=* *9; *p *=* *0.002^d^, paired *t* test; Fig. 6*C*) and initially reduced the CV of the baseline ISIs (control: 0.035; SD, 0.023; NS309: 0.020; SD, 0.010; *p *=* *0.02^e^, paired *t* test; Fig. 6*D*). However, in some cells NS309 eventually disrupted pacemaking, causing missed beats during which spiking was replaced by a subthreshold oscillation (Fig. 6*E*). Because this phenomenon is not compatible with PRC analysis, which requires rhythmic firing, the data used for PRC measurement were limited to the period of NS309 wash-in before missed beats were observed. The average effect of NS309 on the PRC is shown in Figure 6*F*. NS309 reduced sensitivity at all phases of the ISI, but the effect was largest at early phases, consistent with an enhanced SK conductance activated during and/or shortly before each action potential and decaying across the ISI. This increased conductance presumably acts to shunt the external current input, reducing its ability to alter the ISI length. On average, NS309 reduced the PRC mean from 0.081 cycles/pA/s (SD, 0.051) to 0.028 cycles/pA/s (SD, 0.015; *p *=* *0.006^f^, paired *t* test; Fig. 6*G*) and shifted the center of mass from 0.51 (SD, 0.05) to 0.65 (SD, 0.12; *p *=* *0.003^g^, paired *t* test; Fig. 6*H*).

**Figure 7.**
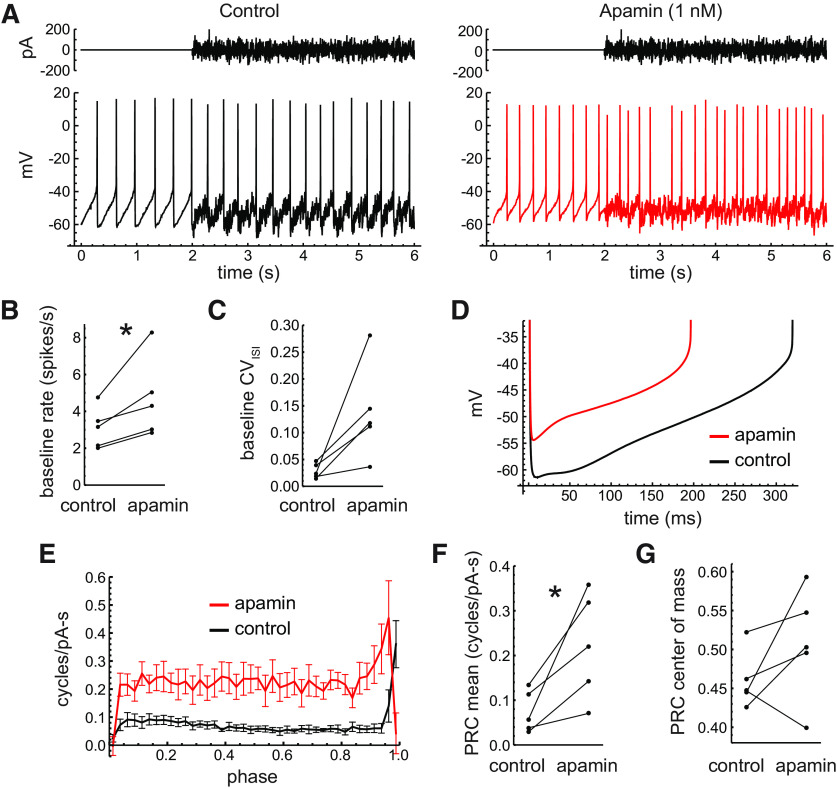
Effects of the partial block of SK channels. ***A***, Pacemaking of an example neuron in control ACSF and in the presence of 1 nm apamin. The example traces were obtained with the same noise stimulus (top) in both conditions. ***B***, Mean baseline firing rates in control ACSF and with apamin (*p *<* *0.05, paired *t* test). ***C***, CV of baseline ISIs in control ACSF and with apamin. ***D***, Average interspike *V*_m_ trajectories of the example cell in control ACSF (black line) and with apamin (red line). Apamin reduced both fast and medium components of the AHP. ***E***, Average PRCs in control ACSF (black) and with apamin (red). Error bars indicate the SEM. ***F***, Mean PRC values in control ACSF and with apamin. ***G***, PRC center of mass in control ACSF and with apamin.

As a second experiment to investigate the effects of the SK channel conductance, we applied the selective blocker apamin. A subsaturating concentration of 1 nm was chosen because it reduced the AHP and increased the firing rate but did not cause burst firing, which is often observed with higher concentrations and would preclude PRC analysis. An example of the effect of apamin on firing at baseline and during noise perturbation is shown in Figure 7*A*. Apamin increased the baseline firing rates from 3.09 spikes/s (SD, 1.12) to 4.70 spikes/s (SD, 2.21; *n *=* *5; *p *=* *0.04^h^, paired *t* test; Fig. 7*B*). Apamin also increased the baseline CV_ISI_ in each cell tested (control: 0.028; SD, 0.014; apamin: 0.138; SD, 0.090), but this effect did not reach significance (*p *=* *0.051, paired *t* test; Fig. 7*C*).

Examination of the average interspike *V*_m_ trajectories (Fig. 7*D*) suggested that, in addition to its direct effect on the SK channel-mediated medium AHP, which is typically apparent as a hyperpolarization reaching a minimum *V*_m_ tens of milliseconds after an action potential, apamin also reduced a faster component of the AHP representing the final phase of action potential repolarization. This effect is likely secondary to increased sodium and potassium channel inactivation produced by the higher overall interspike *V*_m_, which typically reduces both action potential height and fast AHP depth.

Based on the contribution of SK channels to the medium AHP, as well as the effects of NS309 described above, we expected that apamin would affect the PRC most strongly at early phases of the ISI. However, our data showed that apamin greatly increased the PRC across all phases except for the rightmost point where the control PRCs peaked (Fig. 7*E*). On average, apamin increased the PRC mean from 0.075 cycles/pA/s (SD, 0.047) to 0.222 cycles/pA/s (SD, 0.120; *p *=* *0.03^i^, paired *t* test; Fig. 7*F*) but did not change the center of mass (control: 0.46; SD, 0.04; apamin: 0.51; SD, 0.07; *p *=* *0.22, paired *t* test; Fig. 7*G*). There are two general explanations for the large effects of apamin (and, to a lesser extent, NS309) extending to late phases despite SK channels acting primarily to produce the AHP at early phases. First, SK channels may normally be activated across the entire ISI by the levels of calcium present in some compartments of the neuron during pacemaking (Wilson and Callaway, 2000). Second, the AHP produced by SK channels may indirectly affect the sensitivity at later phases by altering the availability of other conductances that are activated later in the firing cycle.

### Effects of an artificial AHP conductance

To investigate whether a defined AHP conductance active early but not late in the ISI could affect the PRC at late phases, we applied an artificial AHP conductance to real DA neurons using the dynamic clamp technique (see Materials and Methods). The artificial AHP was incremented by 4 nS upon the detection of each spike, decayed with a time constant of 50 ms (thereby becoming small within the first half of a typical ISI), and had a reversal potential of −90 mV. Episodes of noise stimulation with and without the artificial AHP were alternated.

**Figure 8.**
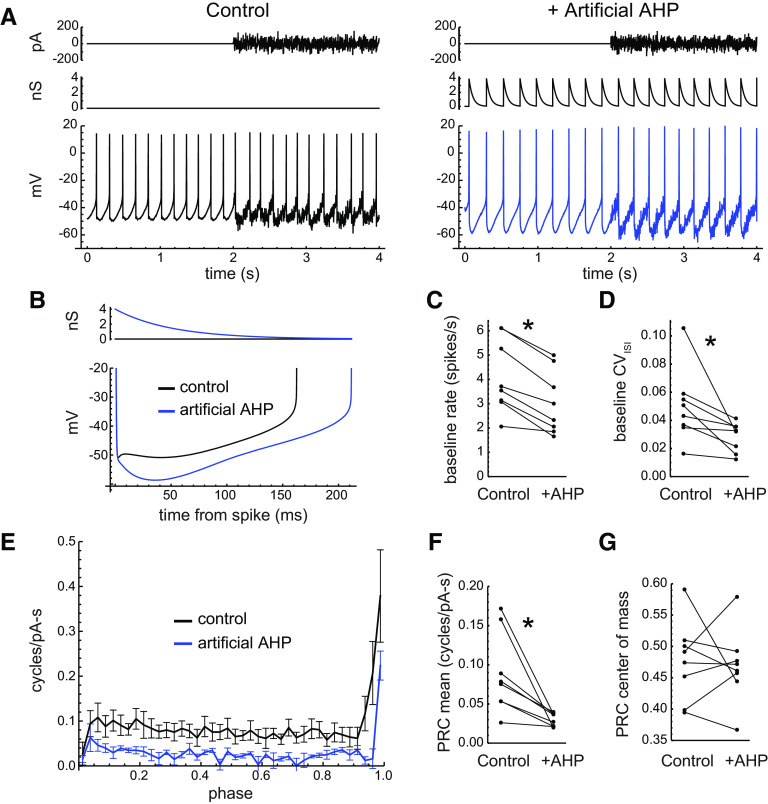
Effects of an artificial AHP conductance. ***A***, Pacemaking of an example neuron in control conditions (left) and with the artificial AHP (right). The top trace is the noise stimulus, and the middle right trace shows the added AHP conductance triggered by each spike. ***B***, Average interspike *V*_m_ trajectories of the example cell without and with the artificial AHP. The top panel shows the time course of the artificial AHP conductance. ***C***, Mean baseline firing rate of each cell without and with the artificial AHP (*p *<* *0.05, paired *t* test). ***D***, CV of baseline ISIs without and with the artificial AHP. ***E***, Average PRCs without and with the artificial AHP, showing a large reduction of sensitivity at early and late phases. ***F***, Mean PRC values without and with the artificial AHP. ***G***, PRC center of mass without and with the artificial AHP.

The effect of the artificial AHP on firing is illustrated in Figure 8*A*. The added spike-triggered conductance deepened the medium AHP (Fig. 8*B*), slowed the firing rate, and reduced the CV of baseline ISIs. The baseline firing rates were lowered from 4.13 spikes/s (SD, 1.51) to 3.04 spikes/s (SD, 1.31; *n *=* *8; *p *=* *0.002^j^, paired *t* test; Fig. 8*C*), and the CVs decreased from 0.050 (SD, 0.026) to 0.028 (SD, 0.010; *p *=* *0.03^k^, paired *t* test; Fig. 8*D*). Surprisingly, although the added AHP conductance decayed almost completely within each ISI, it greatly reduced the PRC across early and late phases (Fig. 8*E*). The mean PRC values decreased from 0.088 (SD, 0.051) to 0.030 (SD, 0.009; *p *=* *0.013^l^, paired *t* test; Fig. 8*F*), while the PRC center of mass showed no significant change (Fig. 8*G*). Overall, the effects of the artificial AHP were opposite to those of apamin and resembled those of NS309. The artificial AHP data showed that a change in the medium AHP can have secondary effects that alter sensitivity at later phases.

### Effects of Kv4 channels

**Figure 9.**
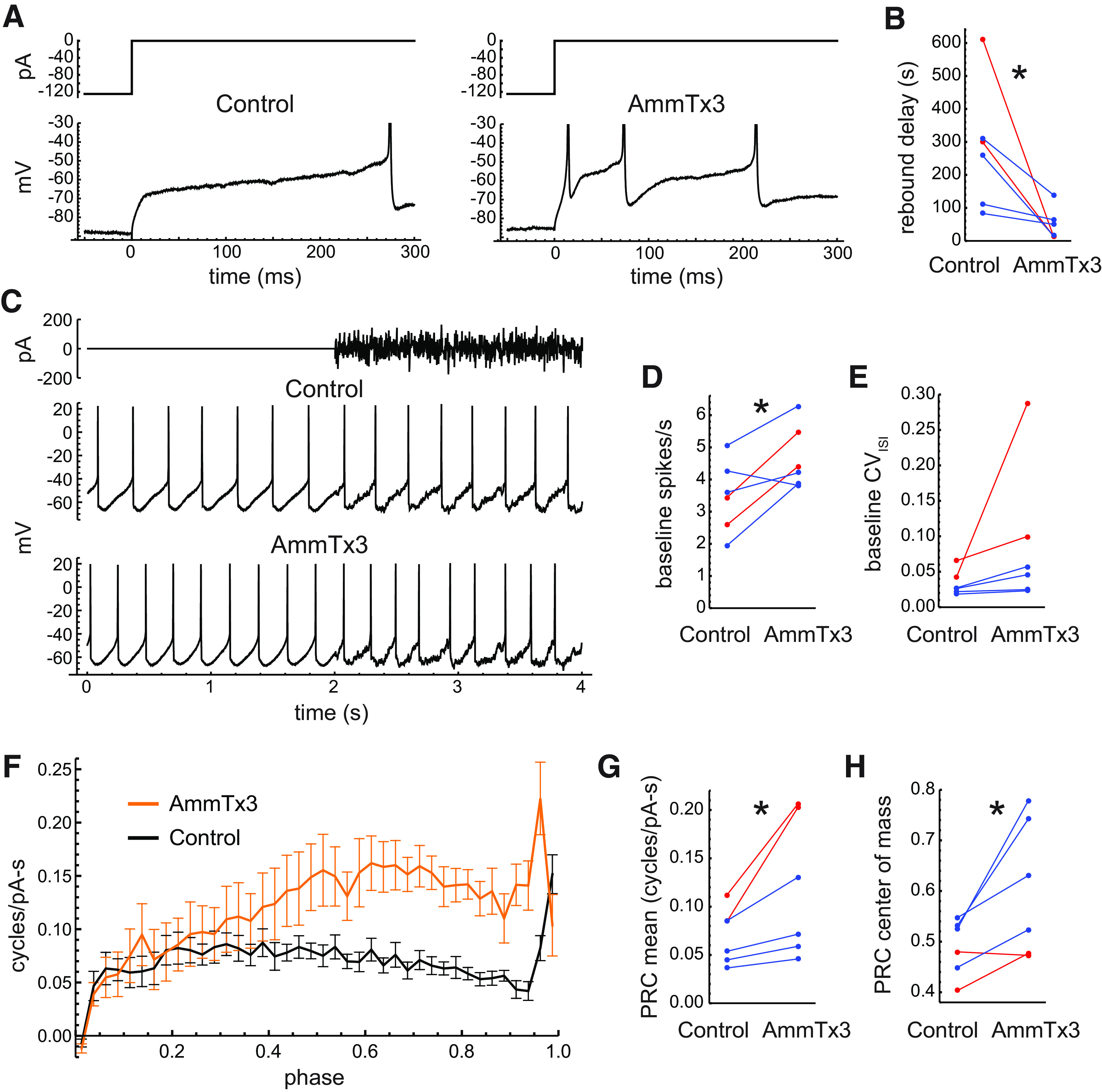
Effects of partial block of Kv4 channels. ***A***, Effect of AmmTx3 (200 nm) on the rebound delay after a 1 s hyperpolarizing current step. The long delay and ramp-like *V*_m_ trajectory seen in control ACSF (left) were eliminated in the presence of AmmTx3 (right). ***B***, Effect on AmmTx3 on the maximum rebound delay of each neuron, measured as the time to the first spike after the end of a −200 pA, 1 s step. For all panels, blue symbols indicate cells tested with 100 nm AmmTx3, and red symbols indicate 200 nm. ***C***, Pacemaking of an example neuron in control ACSF and in the presence of AmmTx3 (100 nm). The control and AmmTx3 data shown were obtained with the same noise waveform. ***D***, Mean baseline firing rates in control ACSF and with AmmTx3 (*p *<* *0.05, paired *t* test). ***E***, CV of baseline ISIs in control ACSF and with AmmTx3. ***F***, Average PRCs in control ACSF and with AmmTx3. Error bars indicate SEM. ***G***, Mean PRC values in control ACSF and with AmmTx3. ***H***, PRC center of mass in control ACSF and with AmmTx3.

In the later portion of the ISI, the major hyperpolarizing conductance in midbrain DA neurons shifts from SK to the A-type potassium conductance mediated primarily by Kv4 channels (Liss et al., 2001; Khaliq and Bean, 2008; Tarfa et al., 2017; Haddjeri-Hopkins et al., 2021). To determine the effects of the Kv4 conductance on the PRC, we applied the scorpion toxin AmmTx3 at 100–200 nm, a concentration range expected to produce partial block of Kv4 channels (Vacher et al., 2002; Amendola et al., 2012; Maffie et al., 2013; Pathak et al., 2016). The best characterized effect of Kv4 channels in DA neurons is a long delay to the first spike after offset of a hyperpolarizing current. Consistent with previous studies using selective toxins to inhibit Kv4 channels (Liss et al., 2001; Khaliq and Bean, 2008; Haddjeri-Hopkins et al., 2021), AmmTx3 greatly reduced this rebound delay (Fig. 9*A*,*B*). On average, the delay was 279 ms (SD, 190) in control ACSF and 49 ms (SD, 49) in the presence of the toxin (*n *=* *6, *p *=* *0.04^m^).

An example of the effects of AmmTx3 on pacemaking at baseline and during noise perturbation is shown in Figure 9*C*. In addition to its effect on firing rate, AmmTx3 replaced the ramp-like portion of each interspike *V*_m_ trajectory with a faster curve upward toward the next action potential, presumably reflecting the reduced rebound delay mechanism. AmmTx3 increased the baseline firing rates from 3.49 spikes/s (SD, 1.12) to 4.67 spikes/s (SD, 0.99; *n *=* *6; *p *=* *0.03^n^, paired *t* test; Fig. 9*D*). AmmTx3 also increased the baseline CV_ISI_ in each cell tested (control: 0.033; SD, 0.018; AmmTx3: 0.090; SD, 0.101), but this effect was not significant (*p *=* *0.20, paired *t* test; Fig. 9*E*). The major effect of AmmTx3 on the PRC was increased sensitivity at middle to late phases, except for the last point (Fig. 9*F*). AmmTx3 increased the PRC mean (control: 0.070 cycles/pA/s; SD, 0.029; AmmTx3: 0.119 cycles/pA/s; SD, 0.072; *p *=* *0.046°, paired *t* test; Fig. 9*G*) and shifted the center of mass from 0.49 (SD, 0.06) to 0.60 (SD, 0.13; *p *=* *0.03^p^, paired *t* test; Fig. 9*H*).

Based on a previous study using voltage-clamp ramp protocols to simulate the interspike membrane potential trajectory in VTA DA neurons (Khaliq and Bean, 2008), we would predict that the active Kv4 conductance is largest in the middle to late portion of the ISI. Our data show increased PRC values at corresponding phases, suggesting that the Kv4 conductance acts directly to shunt external current inputs arriving at these phases, limiting the changes in spike time. In this way, the Kv4 conductance appears to fill the gaps between the periods of high SK conductance during the AHPs. On average, this results in an almost constant, low sensitivity to external input across nearly all phases.

## Discussion

The results of this study establish the PRC as a fundamental, experimentally tractable measure of the input–output relationships of DA neurons during pacemaking. Additionally, we demonstrated that two of the channel classes involved in pacemaking, SK and Kv4, also have major effects on the input sensitivity of these neurons. The moving equilibrium potential established by these and other conductances causes brief membrane potential perturbations to decay rapidly and almost completely toward the natural trajectory. This greatly limits the sensitivity of DA neurons across most of the ISI, making these cells much less sensitive to synaptic inputs compared with other cells such as the local GABA neurons. The combination of conductances expressed by DA neurons produces, on average, nearly constant sensitivity across most of the firing cycle. In this sense, the sampled population of pacemaking DA neurons functions as a nearly perfect integrator, making its responses largely independent of input timing except when action potentials are produced with short latency. When DA neurons are driven to fire at higher rates, they become more sensitive, likely because a more depolarized interspike trajectory inactivates channels such as Kv4 that normally limit sensitivity. An increase in sensitivity at higher rates may bestow DA neurons with the ability to encode input signals more efficiently during episodes of fast firing associated with behaviorally significant events.

### Limitations

The major limitations of using the PRC to describe neuronal input–output relationships are that (1) an estimate of the infinitesimal PRC strictly applies only to small-amplitude input, (2) the noise current we applied as an experimental perturbation does not directly represent synaptic conductances, and (3) input arriving on dendrites may be processed differently from current injected into the soma. However, the second and third limitations are shared with many other widely used measures of neuronal input sensitivity, and considerable evidence suggests that the first limitation is not absolute. Previous studies have shown that experimental PRCs from several cell types successfully predict spike responses to current inputs of substantial amplitude, even when large changes in ISIs are produced (Wilson et al., 2014; Higgs and Wilson, 2017, 2019; Wilson, 2017). For conductance input, the PRC for current can be combined with the natural *V*_m_ trajectory and the reversal potential of the conductance to predict spike timing. Although this approach neglects the deflection of *V*_m_ away from the limit cycle that occurs on perturbation, it is sufficient to provide good predictions of poststimulus time histograms for inhibitory conductances (Simmons et al., 2018, 2020). It is recognized that these predictions may become less accurate as the amplitude of conductance input is increased. Inhibitory inputs can saturate as *V*_m_ is driven toward the reversal potential, and excitatory inputs may also have nonlinear effects. For example, subthreshold excitation of SNc DA neurons by subthalamic afferents causes a phase advance of pacemaking with little effect on the next ISI, whereas suprathreshold excitation causes a short-latency spike and a large shortening of the next ISI (Kang and Futami, 1999). These complexities can only be captured by detailed biophysical models, at the cost of a large expansion of model complexity.

The problem of dendritic input can be addressed in two ways. First, it may be possible to separate the temporal filtering of synaptic current by the dendrites from the pacemaking mechanism represented by the PRC (Goldberg et al., 2007). Second, experimental PRCs can be obtained for real synaptic input or optogenetic input delivered to dendrites. The latter approach has been performed and demonstrates changes in PRC shape consistent with dendritic filtering (Tiroshi and Goldberg, 2019). In SNc DA neurons, glutamate uncaging on dendritic spines during pacemaking was shown to increase spine calcium in a phase-dependent manner, with the largest calcium elevations occurring in the middle-to-late portion of the firing cycle (Hage et al., 2016). This finding demonstrates that specific dendritic mechanisms can produce phase-dependent changes in the response to synaptic input. The impact of these mechanisms on the PRC for excitatory synaptic input remains to be determined.

### Membrane potential responses during pacemaking

Although the DA neurons we recorded all fired rhythmically, their *V*_m_ responses to brief perturbations resembled those of a resting neuron with a short membrane time constant, rapidly decaying almost to the natural trajectory. In fact, the *V*_m_ relaxations were much faster during pacemaking than when the same neurons were held at rest. This observation contrasted with previous data from subthalamic neurons, in which *V*_m_ perturbations across most of the firing cycle show little decay (Farries et al., 2010). The local GABA neurons encountered in the present study appear to occupy an intermediate position on the spectrum, showing *V*_m_ responses with less decay compared with DA neurons, particularly in the later portion of the firing cycle.

In general, the membrane potential dynamics of a pacemaking neuron can be understood in terms of fast–slow analysis, where a neuron is said to have an instantaneous current–voltage (*I–V*) relationship defined by specified values of the slowly changing state variables. Our *V*_m_ perturbation data suggest that the natural trajectories of DA neurons closely track a stable fixed point or upward zero-crossing in the instantaneous *I–V* curve, toward which the perturbed *V*_m_ is attracted. During the ISI, on a fast timescale the DA neuron is essentially resting, but on a longer timescale, as the slow variables change, it is pacemaking. This idea is in contrast to descriptions in which the small d*V*_m_/d*t* across most of the firing cycle is assumed to require exquisite fine-tuning of the balance between inward and outward currents. Instead, the membrane conductance during pacemaking appears to be relatively large compared with a hyperpolarized resting state, and slow pacemaking is robust to a variety of pharmacological manipulations. The reason that d*V*_m_/d*t* is small is that *V*_m_ closely tracks the equilibrium potential.

### How can DA neuron pacemaking be perturbed?

If a pacemaking neuron is attracted toward a stable fixed point determined by slow gating variables, its phase in the firing cycle (defined in theory by the time to the next spike in the absence of further perturbation) can only be altered by changing a slow variable. At least two slow variables are recognized as important for DA neuron pacemaking: intracellular calcium and Kv4 inactivation. During baseline pacemaking, calcium is thought to enter primarily toward the end of the interspike trajectory and during the action potential (Guzman et al., 2009), and, thus, intracellular calcium levels may be resistant to small *V*_m_ changes across most of the ISI. However, intracellular calcium signals produced by dendritic NMDA receptor activation are enhanced during periods of increased firing rate when interspike trajectories are depolarized and the activation of voltage-gated calcium channels is increased (Hage and Khaliq, 2015), and these calcium signals might influence the spike time changes caused by dendritic excitation. At baseline firing rates, Kv4 inactivation may be the major slow variable that can respond to *V*_m_ perturbations. In addition, Kv1 channels can also affect pacemaking in DA neurons in SNc (Guzman et al., 2009) and the VTA (Yee et al., 2022). But how can brief perturbations alter Kv channel inactivation to change the phase of a neuron?

When *V*_m_ is deflected upward from the natural trajectory, Kv4 and Kv1 channels activate rapidly (mainly at late phases, because of their relatively high activation voltages) and inactivate with a slower time course. The degree of additional inactivation caused by the perturbation depends on the amplitude of the *V*_m_ change, the rate of *V*_m_ decay, and the voltage dependence and kinetics of channel inactivation. For a given *V*_m_ perturbation, the resulting change in inactivation is predicted to be greater when *V*_m_ is near the midpoint of the steady-state inactivation curve. For Kv4 channels, which inactivate at relatively hyperpolarized levels (Haddjeri-Hopkins et al., 2021), this suggests that the ability to perturb inactivation may be greater during the early portion of the ISI. This mechanism could potentially explain why some DA neurons have left-weighted PRCs. In contrast, Kv1 channels in DA neurons appear to have somewhat higher inactivation voltages (Yee et al., 2022), so their inactivation may be perturbed more effectively at later phases.

### DA neuron PRC heterogeneity

Although the average DA neuron PRC was nearly flat except at the earliest and latest phases, individual DA neuron PRCs had a variety of shapes, ranging from left to right weighted. The PRCs also showed a wide range of mean values, indicating a large variation of overall sensitivity. Based on the relationship between mean firing rates and mean PRC values (Fig. 5*A*), ∼30% of the variance in mean sensitivity was associated with differences in pacemaking rates. A small fraction of the variance was explained by differences in cell size quantified by the input capacitance (*r*^2^ = 0.11; data not shown), leaving a large portion of the overall sensitivity variance as well as the variations in PRC shape unexplained.

Our findings showing the important roles of SK and Kv4 channels in determining PRC shape and overall sensitivity suggest that variations in the expression or kinetics of these channels, or changes in their activation secondary to differences in intracellular calcium signaling and membrane potential trajectories, could potentially contribute to PRC heterogeneity. Differences in PRC shape could arise from heterogeneous expression of Kv channels with different inactivation voltages. The inactivation time constant might also play a role in shaping the PRC. Inactivation is generally faster for Kv4 compared with Kv1 but can vary considerably among channels of each type, depending on the accessory subunits present (Rettig et al., 1994; Jerng et al., 2005; Li et al., 2006). Differences in the inward currents driving pacemaking are also likely to influence the PRCs and remain an important topic to be explored.

Although the present study did not aim to map DA neuron PRC heterogeneity across anatomic regions or neuronal subtypes, it is likely that PRCs vary among DA neuron subtypes with different biophysical properties. Most of the reported differences are between DA neurons located in SNc versus the lateral and medial VTA, in the ventral tier versus the dorsal tier of SNc, and with different projection targets (Gantz et al., 2018). For example, SNc neurons have been reported to rely more on L-type calcium current and HCN current for pacemaking compared with VTA neurons (Seutin et al., 2001; Chan et al., 2007; Puopolo et al., 2007; Khaliq and Bean, 2010), and rebound delays governed by the inactivation of A-current are much longer in VTA neurons projecting to the prefrontal cortex and nucleus accumbens compared with SNc neurons projecting to the dorsal striatum (Lammel et al., 2008; Tarfa et al., 2017). We recorded from DA neurons in a small region primarily including the medial SNc but likely including some lateral VTA neurons located near the SNc/VTA border (Fig. 1). We did not observe significant differences in PRC shape or mean sensitivity between the more rostral/lateral portion of our target region (rostral to MT) and the more caudal/medial portion (just medial to MT) or between neurons from the three slices of our preparation (data not shown). Therefore, we interpret our results as representative of the majority of medial SNc neurons.

For anatomic mapping of PRC heterogeneity, it will be necessary to record across a wider range of locations and collect accurate data on cell location, ideally combined with cell identification based on projection targets. With this methodology, the PRC will provide a valuable measure to characterize the heterogeneity of intrinsic input–output properties across the population of midbrain DA neurons. If PRC shapes do vary systematically among subtypes of DA neurons, this could potentially provide a mechanism for differential sensitivity to inputs with specific timing properties, such as the 4 Hz cortical/hippocampal/VTA oscillation described by Fujisawa and Buzsáki (2011).

### The value of insensitivity

The low-sensitivity, stable pacemaking of DA neurons may serve two purposes. First, DA neurons are nodes of convergence for massive excitatory and inhibitory synaptic input from multiple brain regions (Watabe-Uchida et al., 2012), and they function to integrate multiple input streams to evaluate deviations from expectation, leading to modulation of dopamine release across widespread target areas. In this context, allowing single, weak synaptic inputs to strongly affect spike output could obscure the detection of truly salient events. Second, the range of DA neuron output firing rates is relatively limited compared with many other cell types, largely as a consequence of strong sodium channel inactivation (Knowlton et al., 2021), so relatively low sensitivity is necessary to scale the large range of possible synaptic inputs to the available output range. It is unclear whether this limited range is in some way optimal for dopamine neuron function. However, some limit on firing activity may be necessary because of the enormous metabolic demands of supporting the massive axonal arbors exhibited by this cell type (Pissadaki and Bolam, 2013; Pacelli et al., 2015).

The limitation of sensitivity observed during baseline pacemaking may be overcome by strong synaptic inputs that push the neuron completely outside its normal limit cycle and into burst firing, during which AHPs become shallow, Kv4 channels likely inactivate almost completely, and L-type calcium currents and their influence on firing are enhanced by the depolarized interspike trajectories (Hage and Khaliq, 2015; Shin et al., 2022). These changes likely shift the responses to brief *V*_m_ perturbations from restorative to regenerative. Thus, we would predict that the sensitivity to transient inputs will increase along with the firing frequency, providing a larger bandwidth for information transmission by increasing spike time perturbation in the burst firing mode.

### Significance

The results of this study establish the PRC as a tool to examine the input–output properties of DA neurons in the context of their natural pacemaking activity. The PRC relates the input sensitivity of a neuron to the dynamics and ionic mechanisms of pacemaking in an intuitively comprehensible way, as the sensitivity to input arriving at a given phase depends on the conductances active when the input arrives and on other conductances whose later activation is altered by the initial perturbation of the interspike trajectory. As we learn more about the roles of specific ion channels in shaping the PRC, it may become a useful tool to characterize the differences among DA neuron subtypes and to identify biophysical alterations in disease models and under a variety of behavioral and environmental manipulations.

## Synthesis

Reviewing Editor: Masha Prager-Khoutorsky, McGill University Decisions are customarily a result of the Reviewing Editor and the peer reviewers coming together and discussing their recommendations until a consensus is reached. When revisions are invited, a fact-based synthesis statement explaining their decision and outlining what is needed to prepare a revision will be listed below. The following reviewer(s) agreed to reveal their identity: Kauê Costa, Carmen Canavier.

NA
